# A Pragmatic Mapping of Perceptions and Use of Digital Information Systems in Primary Care in Sweden: Survey Study

**DOI:** 10.2196/49973

**Published:** 2023-10-25

**Authors:** Anita Sant’Anna, Jens Nygren

**Affiliations:** 1 Viniam Consulting AB Halmstad Sweden; 2 School of Health and Welfare Halmstad University Halmstad Sweden

**Keywords:** digital information systems, implementation, primary care, health care professionals, information system, information systems, usability, adoption, perception, perceptions, technology use, perspective, perspectives

## Abstract

**Background:**

Electronic health records and IT infrastructure in primary care allow for digital documentation and access to information, which can be used to guide evidence-based care and monitor patient safety and quality of care. Quality indicators specified by regulatory authorities can be automatically computed and presented to primary care staff. However, the implementation of digital information systems (DIS) in health care can be challenging, and understanding factors such as relative advantage, compatibility, complexity, trialability, and observability is needed to improve the success and rate of adoption and diffusion.

**Objective:**

This study aims to explore how DIS are used and perceived by health care professionals in primary care.

**Methods:**

This study used quantitative assessment to gather survey data on the use and potential of DIS in health care in Sweden from the perspectives of primary care personnel in various roles. The digital questionnaire was designed to be short and contained 3 sections covering respondent characteristics, current use of platforms, and perceptions of decision support tools. Data were analyzed using descriptive statistics, nonparametric hypothesis testing, ordinal coefficient α, and confirmatory factor analysis.

**Results:**

The study collected responses from participants across 10 regions of Sweden, comprising 31.9% (n=22) from private clinics and 68.1% (n=47) from public clinics. Participants included administrators (18/69, 26.1%), a medical strategist (1/69, 1.4%), and physicians (50/69, 72.5%). Usage frequency varied as follows: 11.6% (n=8) used DIS weekly, 24.6% (n=17) monthly, 27.5% (n=19) a few times a year, 26.1% (n=18) very rarely, and 10.1% (n=7) lacked access. Administrators used DIS more frequently than physicians (*P*=.005). DIS use centered on quality improvement and identifying high-risk patients, with differences by role. Physicians were more inclined to use DIS out of curiosity (*P*=.01). Participants desired DIS for patient follow-up, lifestyle guidance, treatment suggestions, reminders, and shared decision-making. Administrators favored predictive analysis (*P*<.001), while physicians resisted immediate patient identification (*P*=.03). The 5 innovation attributes showed high internal consistency (α>.7). These factors explained 78.5% of questionnaire variance, relating to complexity, competitive advantage, compatibility, trialability, and observability. Factors 2, 3, and 4 predicted intention to use DIS, with factor 2 alone achieving the best accuracy (root-mean-square=0.513).

**Conclusions:**

Administrators and physicians exhibited role-based DIS use patterns highlighting the need for tailored approaches to promote DIS adoption. The study reveals a link between positive perceptions and intention to use DIS, emphasizing the significance of considering all factors for successful health care integration. The results suggest various directions for future studies. These include refining the trialability and observability questions for increased reliability and validity, investigating a larger sample with more specific target groups to improve generalization, and exploring the relevance of different groups’ perspectives and needs in relation to decisions about and use of DIS.

## Introduction

The wide adoption of electronic health records and general IT infrastructure in primary care facilitates both digital documentation and access to information. This digital infrastructure can be leveraged to guide evidence-based care and help monitor patient safety and quality of care at the individual or group level [1].

Primary care providers must track a number of quality indicators specified by regulatory authorities. These quality indicators are used to monitor and compare the quality of care across local and regional organizational boundaries [2,3]. Thanks to the wide adoption of electronic health records, these indicators can be automatically computed and presented to primary care staff [4]. In Sweden, these indicators are part of a digital platform called “Quality of Primary Care” (swe. Primärvårdskvalitet), to which the vast majority of primary care providers have access [5]. This platform is an example of a digital information system (DIS).

The purpose of DIS in health care is to facilitate the visualization and analysis of the data contained in electronic health records and administrative data both on an individual and population level [6,7]. In addition to presenting information such as quality indicators, these systems may also provide clinical decision support tools. The use of DIS among administrators, doctors, and nurses includes a spectrum of diverse purposes, intricately intertwined with their distinct roles, specialized training, individual preferences, and intrinsic motivations. This multiplicity of factors influences the unique ways in which each group engages with these systems, shaping their interactions, decision-making processes, and overall contributions within the health care ecosystem [8,9]. They may also have distinct perspectives on the value of DIS, both within their daily duties and the clinic’s overall functioning. These viewpoints arise from factors like duties and tasks, desired outcomes, familiarity with technologies, and personal experiences. This variance of perspectives shapes the perception and integration of digital systems, influencing clinical operational efficiency [10-12]. Understanding these different roles and perceptions is essential to developing systems that work well in practice and fulfill users’ needs [13,14].

In general, the introduction or implementation of a new DIS is difficult [12,13]. Oftentimes, they do not add enough value, they are too generic and not aligned with local work processes, or they do not consider practical barriers to implementation [15,16]. It is also unknown how DIS are used in practice, by whom, how often, and to what purpose, as well as how they are perceived and what innovative features DIS users wish to see in the future [17]. The introduction of an innovation such as DIS can be understood and studied as part of a process toward adoption and diffusion. In relation to a specific innovation and the intended setting and context for its introduction, there are several factors that affect the adoption process and the diffusion within and across settings [18]. If such factors are understood they can be targeted to facilitate and improve both the success and rate of adoption and diffusion. In this context, 5 attributes of innovation are decisive—relative advantage, compatibility, complexity, trialability, and observability—and have been respectively defined as “the degree to which an innovation is perceived as being better than the idea it supersedes,” “the degree to which an innovation is perceived as consistent with the existing values, past experiences, and needs of potential adopters,” “the degree to which an innovation is perceived as relatively difficult to understand and use,” “the degree to which an innovation may be experimented with on a limited basis,” and “the degree to which the results of an innovation are visible to others” [18]. In the context of introducing DIS to improve health care, it is therefore of great importance to understand how DIS are perceived by the stakeholders affected by its introduction, with respect to its different attributes and in relation to different intended uses [12].

This study adopts a pragmatic approach using a familiar DIS within primary care to prompt reflection regarding use and potential development and future applications. Consequently, the study aims to explore how DIS are used and perceived by health care professionals in primary care.

## Methods

### Study Design

The study has an exploratory design and is based on quantitative assessment, using descriptive statistics, of survey data collected during April-May 2022 in accordance with the CHERRIES (Checklist for Reporting Results of Internet E-Surveys) [19].

### Participants

We wanted to reach a large number of primary care personnel in various roles. To make it feasible to reach users of DIS we used two approaches for the distribution of a closed web-based survey: (1) directly emailing publicly listed contact persons at primary care clinics when that information was available and (2) requesting an interest organization to distribute the link to the questionnaire to its members or networks. While using this pragmatic approach enabled the creation of a convenience sample comprising a larger respondent count, it constrained our outreach predominantly to administrators and physicians.

From publicly available information on the web, we curated a total of 44 contact emails from primary care health centers in a local region in southern Sweden and 64 contact emails from a private primary care provider across the country. We contacted the 25 regional chapters of The Swedish Association of General Practice as well as the Innovation Platform at Region Västra Götaland and the Digital Well Arena in Region Värmland and asked them to distribute our questionnaire to people working in the primary care sector.

### Data Collection

The digital questionnaire was kept as short as possible so that it would take less than 5 minutes to answer. This was a pragmatic choice intended to maximize the number of respondents in a short period of time. The questionnaire was structured in 3 sections developed for the purpose of the study (Multimedia Appendix 1). It included both self-developed questions of a descriptive nature (sections 1-2) and questions based on a previously validated instrument (section 3). The three sections covered were as follows:

1. Respondent characteristics, including role, whether they work at a private or public provider, and their geographical region (3 questions);
2. Use of current platforms, including access to digital tools, frequency of use, and reason for use (18 questions);
3. Perceptions of decision support tools, including unmet needs, perceptions, and beliefs about future DIS (12 questions).

Most questions were multiple choice on an ordinal scale. Sections 2 and 3 asked about the respondents’ level of agreement or disagreement with a number of statements on a 5-level ordinal scale: completely agree, partially agree, indifferent, partially disagree, or completely disagree. Section 3 was developed based on Roger 5 attributes of innovation [16]—relative advantage, compatibility, complexity, trialability, and observability of results—and used a previously developed survey as template for the development [17]. To assess each of these attributes, 2 statements were developed. In addition, 2 final statements were designed to assess the respondents’ intention to use DIS.

Statements were developed from previous interviews with primary care physicians, primary care administrators, medical experts in the Life Science industry, and technologists developing decision support tools. The full questionnaire is available in Multimedia Appendix 1. The questionnaire was reviewed for face validity by technologists working with clinical decision support tools, life science professionals, and health innovation and implementation researchers. The answers from the first-week respondents (N=13) were used to check that the list of roles was representative and that they were not indifferent to all items. The questionnaire was updated with additional terms for roles in the first question to better reflect what the first respondents had written under the item “other.” No other adjustments were made.

Participants were, during data collection, presented with all questions regardless of their responses to preceding queries. All questions were obligatory, and participants were provided with the alternative to respond “Don’t know” or “Neither.” Participants were allowed to review their responses before submitting the survey. The data were checked for duplicated entries to ensure that participants had not answered the questionnaire twice by accident. The usability and technical functionality of the electronic questionnaire were tested internally before fielding the questionnaire.

### Data Analysis

Questionnaire answers were analyzed with descriptive statistics. Each section was analyzed for differences in answers according to roles (administrators vs physicians). This was done using the Mann-Whitney Wilcoxon Test and a significance level of 0.05, to test the null hypothesis that answers from the 2 different groups follow the same distribution. The test chosen is a nonparametric rank-sum test that has been shown to perform robustly even for small sample sizes of 10 or fewer observations [20,21].

Section 3 of the questionnaire was analyzed for internal consistency, in this case, the level of agreement between items related to the same attribute. This was done using ordinal coefficient α based on a polychoric correlation matrix [22,23].

Confirmatory factor analysis with varimax rotation and 5 factors was also performed on section 3 of the questionnaire to ensure that Roger 5 attributes were indeed captured in the questions. The 2 items related to intention to use presented high internal consistency and were averaged into one factor. The resulting factors were then used to predict the respondent’s intention to use DIS. A linear regression model was trained using 50 randomly selected entries. Model performance was tested with the remaining 20 entries. The accuracy of the model was evaluated visually and with root-mean-square (RMS) errors. All statistical analysis was done using RStudio (version 2023.03.0+386, Posit PBC).

### Ethical Considerations

The study adheres to the principles outlined in the Declaration of Helsinki and fulfilled the following research requirements: information, consent, confidentiality, and participant safety. Ethical approval for the research was not formally required under Swedish law, as no personal or sensitive information was handled. Each participant received written information encompassing the study’s objectives and inception, outlining their role in the study, clarifying the collection of exclusively anonymous data, and delineating the methods for data collection and storage. They were also informed about the voluntary nature of participation, confidentiality, and the option to withdraw their consent at any point, without the need for justification.

## Results

We received a total of 70 responses across 10 of the 21 regions of Sweden (Table 1). Of the 69 respondents informing on their workplace and role, 31.9% (n=22) worked at private clinics and 68.1% (n=47) worked at public clinics and, 26.1% (n=18) worked as administrators or head of operations, 1.4% (n=1) had the role as a medical strategist, and the remaining 72.5% (n=50) were physicians. Of the respondents, 11.6% (n=8) used DIS on a weekly basis, 24.6% (n=17) used DIS on a monthly basis, 27.5% (n=19) used DIS a few times a year, 26.1% (n=18) used DIS very rarely, and 10.1% (n=7) said they did not have access to DIS. However, the frequency of use differed significantly by role. Administrators were more likely to use DIS more frequently than physicians (*P*=.005; Table 2).

**Table 1:** Regional distribution of respondents (N=70).

Region	Respondents, n (%)
Kronoberg	17 (24.3)
Halland	17 (24.3)
Skåne	12 (17.1)
Västra Götaland	8 (11.4)
Uppsala	6 (8.6)
Blekinge	5 (7.1)
Gotland	2 (2.9)
Kalmar	1 (1.4)
Gävleborg	1 (1.4)
Dalarna	1 (1.4)

**Table 2:** Frequency of use depending on participants’ roles (N=68; 2 participants did not inform on both what their role was and frequency of use and were excluded from this table).

	Administrators (N=18), n (%)	Physicians (N=50), n (%)
No access to digital information systems	0	7 (14.0)
Rarely	2 (11.1)	16 (32.0)
Yearly	5 (27.8)	14 (28.0)
Monthly	9 (50.0)	8 (16.0)
Weekly	2 (11.1)	5 (10.0)

**Figure 1:**
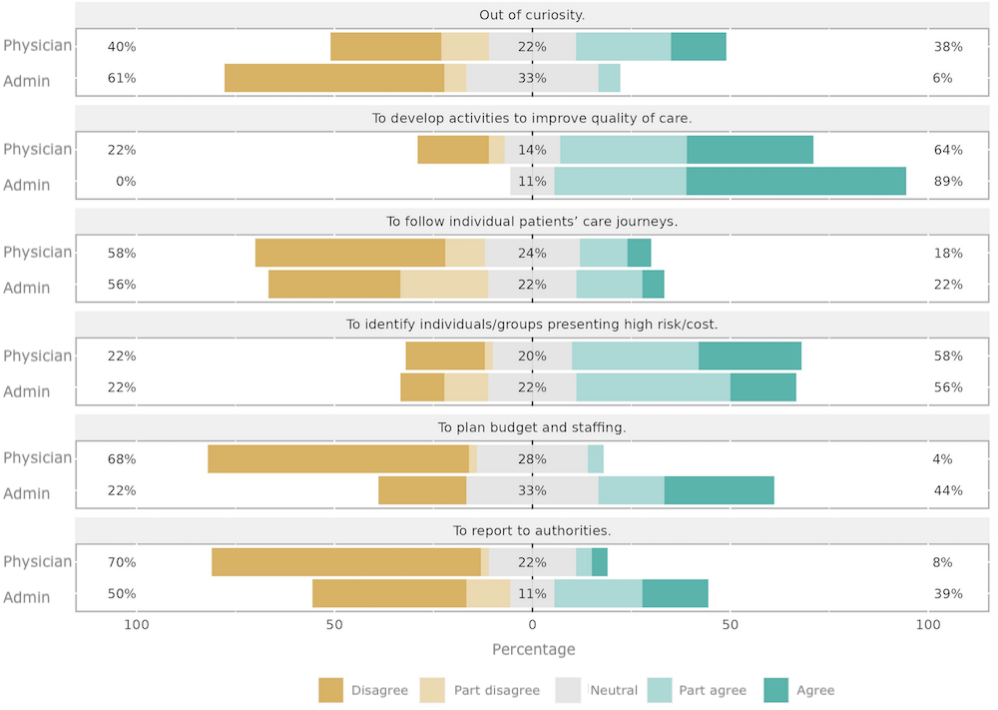
Participants’ responses to statements on their use of digital information systems in their everyday practice in response to the question “How well do the following statements agree with why you use the digital tool today?”.

In general, a large portion of participants indicated that DIS are not used to follow individual patients’ care journeys. On the other hand, a large portion of participants indicated that DIS are used for guiding quality improvement activities and for identifying high-risk or high-cost patients. Differences in the use of DIS were aligned with specific roles. Administrators were significantly more likely than physicians to use DIS for reporting to authorities (*P*=.01), for developing activities to improve quality of care (*P*=.02), and for planning budget and staffing (*P*<.001). On the other hand, physicians were significantly more likely than administrators to use DIS out of curiosity (*P*=.01; Figure 1).

**Figure 2:**
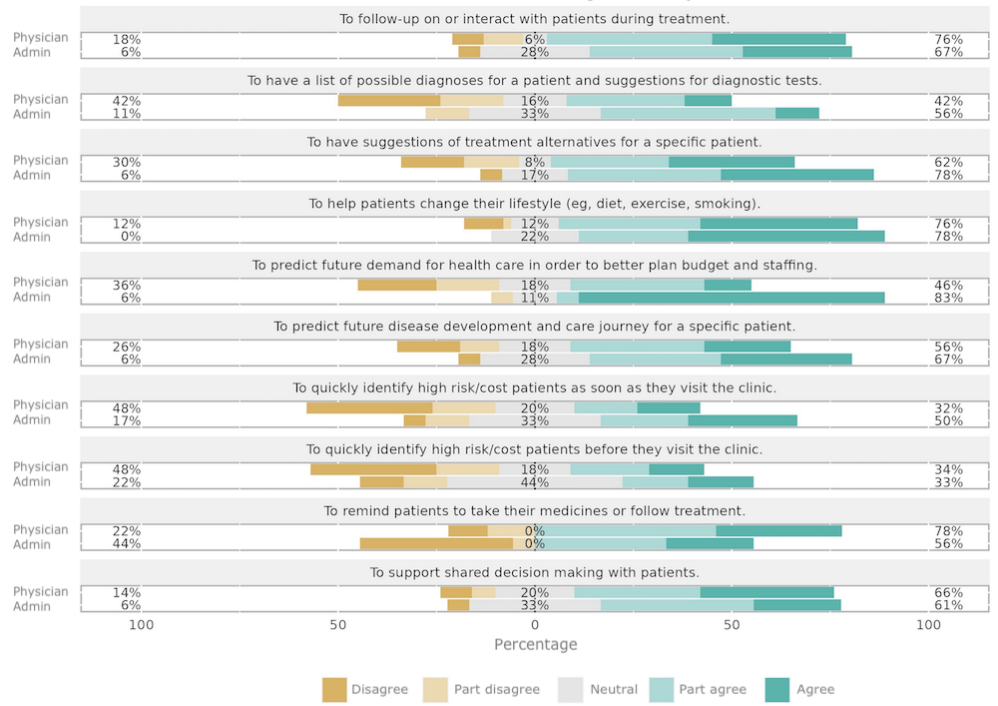
Participants’ statements on their use of digital information systems in their everyday practice in response to the question “How well do the following statements agree with your needs?”.

In general, participants indicated that they would like to have DIS to support the follow-up of patients, lifestyle changes for patients, suggestions for treatment options, and reminders for patients to follow treatment, as well as making shared decisions with patients. None of the suggested future developments were seen as negative by either of the roles. However, there were a few areas where administrators and physicians differed in their opinion (Figure 2). For example, administrators were more positive toward predictive analysis of care needs to facilitate planning of budget and staffing (*P*<.001). Physicians on the other hand were more negative toward identifying high-risk or high-cost patients as soon as they visit the clinic (*P*=.03).

Section 3 of the questionnaire included 2 items for each of Roger 5 attributes of innovation: competitive advantage, complexity, compatibility, trialability, and observability. The answers to each attribute were evaluated for internal consistency using the ordinal reliability coefficient α. Starting from the assumption that several items measure the same latent variable, the reliability coefficient indicates how consistent those items are as a group. An α above .9 is considered excellent, whereas an α between .7 and .8 is considered acceptable. Items related to competitive advantage (α=.94), complexity (α=.96), and compatibility (α=.90) presented excellent internal consistency. Trialability (α=.73) and observability (α=.72) presented acceptable internal consistency, indicating a certain dissonance between the 2 items related to these 2 attributes. The additional 2 items referring to intention to use also presented excellent internal consistency (α=.93).

The answers to section 3 of the questionnaire were decomposed into 5 factors to test the hypothesis that these questions capture the 5 innovation attributes. Indeed, results show that the resulting 5 factors explain 78.5% of the variance in the data. In addition, each resulting factor was highly linked to one of the five attributes: competitive advantage 1 and 2 (0.090 and 0.247), complexity 1 and 2 (0.005 and 0.218), compatibility 1 and 2 (0.094 and 0.318), trialability 1 and 2 (0.550 and 0.005), and observability 1 and 2 (0.618 and 0.005). We may therefore refer to factor 1 as mostly representing complexity, factor 2 as representing competitive advantage, factor 3 as representing compatibility, factor 4 as representing trialability, and factor 5 as representing observability. For trialability and observability, loadings were high for 1 item but relatively low for the other item. This is consistent with the slightly lower ordinal reliability coefficient for those items, and the high values of uniqueness of variables trialability 1 and observability 1 (Table 3). This indicates that these variables contain unique information that does not conform with the 5 resulting factors.

**Figure 3:**
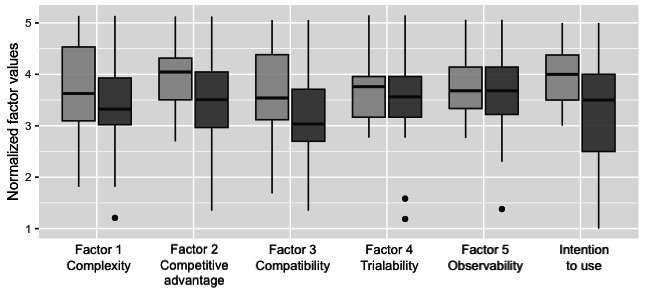
Perceptions decision support of digital information systems by role, administrators (light gray) and physicians (dark gray).

Administrators were significantly more positive than doctors about DIS being compatible with their work processes (*P*=.04). In addition, administrators were significantly more positive than physicians in their intention to use DIS (*P*=.01). Intention to use did not significantly differ between respondents who used DIS frequently (ie, weekly or monthly) and the remaining respondents (*P*=.30; Figure 3).

All factors are positively correlated with the intention to use. Pearson correlation coefficient was highest for factor 2 (*r*=0.85), followed in order by factor 3 (*r*=0.78), factor 5 (*r*=0.74), factor 4 (*r*=0.73), and factor 1 (*r*=0.64). Intention to use was predicted from the 5 factors, starting with factor 2, which had the highest correlation, and consecutively adding more factors in order of correlation. RMS error was used as a measure of accuracy for each model. The best accuracy was obtained using factor 2 only (RMS=0.513), followed by a model using all 5 factors (RMS=0.524). Accuracy results were slightly worse for a model using factors 2 and 3 (RMS=0.523), followed by a model including factors 2, 3, 4, and 5 (RMS=0.532). The worst-performing model included factors 2, 3, and 5 (RMS=0.533).

**Table 3:** Factor loadings of questions in relation to 5 innovation attributes (bold values indicate items with high faction loadings).

Loadings	Factor 1	Factor 2	Factor 3	Factor 4	Factor 5
Competitive advantage 1	0.158	0.882^a^	0.259	0.170	0.108
Competitive advantage 2	0.224	0.709^a^	0.390	—^b^	0.213
Complexity 1	0.956^a^	—	0.246	0.133	—
Complexity 2	0.820^a^	0.160	0.261	0.125	—
Compatibility 1	0.359	0.342	0.785^a^	0.208	—
Compatibility 2	0.425	0.286	0.632^a^	—	0.124
Trialability 1	0.272	0.404	0.131	0.378^a^	0.230
Trialability 2	0.184	0.182	0.140	0.929^a^	0.213
Observability 1	–0.139	0.450	—	0.246	0.310
Observability 2	—	0.259	—	0.222	0.934^a^

^a^Values indicate items with high faction loadings.

^b^Not available.

## Discussion

### Principal Findings

In this study, we applied an approach to broadly reach out to users of DIS at a national level. A digital survey with questions about the participants’ characteristics and demographics, current use, and perceptions of DIS was distributed through emails directly to potential participants and through email lists and networks. A limited number of responses were received, but still sufficient to address the study’s research questions. One-third of the participants held roles as administrators and two-thirds as physicians. Almost two-thirds used DIS often or a few times a year, while approximately one tenth had no access to DIS at all, and thereby responded to the questionnaire based on perceptions rather than own experiences. The administrators used the DIS more often and both the administrators’ and the physicians’ use followed purposes based on their professional roles, that is, more administrative purposes and more clinical purposes respectively.

Both roles were very positive about using DIS to improve the quality of care and to identify patients and groups at high risk and high costs. In cases where there was a disinterest in the use of DIS, it was associated with the application not being linked to the professional role or work tasks. For example, administrators were less interested in using DIS out of pure curiosity, while physicians were less interested in applying DIS for budget work and staffing. The only application area that was seen as predominantly negative for both roles was to follow individual patients’ care pathways. Given the value for both administrators, regarding risk assessment and cost calculation; and doctors, regarding opportunities to improve quality in clinical decisions and care planning, one could expect more positive attitudes toward this purpose of use. Whether this lower interest was due to uncertainty about the meaning of applying DIS to follow individual patients’ care pathways, or that, for example, the application was deemed not to be consistent with work tasks or the professional roles of the participants, remains to be investigated. Overall, these findings align with previous research that the attitudes toward using DIS in health care are intricately shaped by a multitude of factors, such as the application area, the professional role, and work tasks. These factors include not only the specific application domains but also the distinct professional roles and inherent tasks of individuals within the health care context [8,9,12]. The findings from this study and existing knowledge underscore the interplay between contextual variables and illustrate the essential role they play in shaping the attitudes and inclinations toward integrating DIS within health care settings [10,12]. To promote the use of DIS in health care, this study and other research in this field [18,24] suggest it may be important to consider these factors and tailor the application of DIS to the needs of different stakeholders.

In all contexts of the introduction of new innovations, stakeholders’ perceptions of the innovation have a decisive role in the innovation’s successful adoption and diffusion within the organization, and to other similar application areas in other organizations [18]. In order to succeed in introducing DIS in clinical organizations with the aim of improving health care, it is thus important to understand how DIS is perceived by the stakeholders affected by its introduction. To investigate this, we developed a self-assessment instrument for stakeholders’ perceptions of DIS based on Roger 5 innovation attributes [18]. The instrument items were constructed based on a previous study of attitudes toward the integration of social media in university education [25]. The construction of 2 items to represent each innovation attribute was validated through an assessment of their internal consistency and factor loadings in relation to the comprehensive spectrum of 5 attributes. The subsequent correlation analysis, linking participant responses with their intentions to use DIS, unveiled a noteworthy positive alignment between higher-rated perceptions of DIS’s innovation attributes and an elevated intention to engage with the system. This suggests that a swift preliminary evaluation of stakeholders’ favorable attitudes and use intentions can be achieved by evaluating the perceived relative advantage of DIS. However, to attain a deeper and more nuanced understanding, it is prudent to holistically examine all 5 of Roger’s proposed innovation adoption factors in conjunction. This approach ensures a comprehensive exploration of the different dynamics influencing stakeholders’ inclinations to adopt DIS based on their perceived attributes and attitudes and could facilitate a more informed and contextually sensitive evaluation of their intentions.

### Limitations

The study faced a challenge in terms of reaching a sufficient number of participants, and the response rate was relatively low. The findings may thus not fully represent the views and experiences of the entire population of interest, and there could be some sampling bias. More than half of the respondents reported using DIS often. While this may reflect the growing trend of digitalization in health care, it could also mean that the sample was overrepresented with participants with positive experiences and attitudes toward DIS and not representative of the general population, which may include individuals who have limited access or skills in using digital technologies. The study measured the intention to use DIS among both users and nonusers. However, this approach could introduce some variability in the results, as those who have prior experience with such systems may have different motivations and expectations compared to those who have only a perception or awareness of these systems. Therefore, the findings should be interpreted with caution and may not apply to all potential users.

### Conclusions

In conclusion, this study used a comprehensive approach to engage a diverse range of DIS users at a national level. The survey responses provided valuable insights despite the limited number received, addressing key research questions. Administrators and physicians demonstrated varying patterns of DIS use aligned with their professional roles, reflecting distinct attitudes toward different application areas. Notably, DIS was positively embraced for quality enhancement and identifying high-risk patients, with concerns arising mainly in relation to following individual care pathways. Stakeholders’ perceptions of DIS, influenced by attributes such as professional roles and application contexts, underscore the need for tailored approaches in promoting DIS adoption within health care. By drawing from Roger’s innovation attributes, this study developed an assessment instrument to gauge stakeholder perceptions, revealing a correlation between positive perceptions and intention to use DIS. Considering all innovation factors is essential for a comprehensive understanding of stakeholders’ attitudes toward DIS integration, offering valuable insights for successful adoption and diffusion in health care settings. Based on the results, there are several potential avenues for future studies. For example, the trialability and observability questions could be refined to improve their reliability and validity. Additionally, a larger sample with more defined target groups could be investigated to enhance the generalization of the findings. Finally, more clearly defined relevance of the groups with respect to decisions about and use of DIS could be explored to gain a deeper understanding of their perspectives and needs.

## Appendix

Multimedia Appendix 1 Digital questionnaire developed for the purpose of the study.

## Abbreviations

CHERRIES: Checklist for Reporting Results of Internet E-Surveys
DIS: digital information systems
RMS: root-mean-square

## Data Availability

The data sets generated or analyzed during this study are available from the corresponding author upon reasonable request.

## References

[1] Atasoy H, Greenwood BN, McCullough JS (2019). The digitization of patient care: a review of the effects of electronic health records on health care quality and utilization. Annu Rev Public Health.

[2] Cinaroglu S, Baser O (2016). Understanding the relationship between effectiveness and outcome indicators to improve quality in healthcare. Total Qual Manag Bus Excell.

[3] Horvat A, Filipovic J (2018). Healthcare system quality indicators: the complexity perspective. Total Qual Manag Bus Excell.

[4] Essén A, Scandurra I, Gerrits R, Humphrey G, Johansen MA, Kierkegaard P, Koskinen J, Liaw ST, Odeh S, Ross P, Ancker JS (2018). Patient access to electronic health records: differences across ten countries. Health Policy Technol.

[5] Sveriges Kommuner och landsting (SKL) Primärvårdskvalitet—ett stöd för kvalitetsarbete i primärvården.

[6] Saigí-Rubió F, Pereyra-Rodríguez JJ, Torrent-Sellens J, Eguia H, Azzopardi-Muscat N, Novillo-Ortiz D (2021). Routine health information systems in the European context: a systematic review of systematic reviews. Int J Environ Res Public Health.

[7] Epizitone A, Moyane SP, Agbehadji IE (2022). Health information system and health care applications performance in the healthcare arena: a bibliometric analysis. Healthcare (Basel).

[8] Ahmadian L, Dorosti N, Khajouei R, Gohari SH (2017). Challenges of using hospital information systems by nurses: comparing academic and non-academic hospitals. Electron Physician.

[9] Abdekhoda M, Ahmadi M, Gohari M, Noruzi A (2015). The effects of organizational contextual factors on physicians' attitude toward adoption of electronic medical records. J Biomed Inform.

[10] Marwaha JS, Landman AB, Brat GA, Dunn T, Gordon WJ (2022). Deploying digital health tools within large, complex health systems: key considerations for adoption and implementation. NPJ Digit Med.

[11] Moore EC, Tolley CL, Bates DW, Slight SP (2020). A systematic review of the impact of health information technology on nurses' time. J Am Med Inform Assoc.

[12] Petersson L, Larsson I, Nygren JM, Nilsen P, Neher M, Reed JE, Tyskbo D, Svedberg P (2022). Challenges to implementing artificial intelligence in healthcare: a qualitative interview study with healthcare leaders in Sweden. BMC Health Serv Res.

[13] Nilsen P, Seing I, Ericsson C, Birken SA, Schildmeijer K (2020). Characteristics of successful changes in health care organizations: an interview study with physicians, registered nurses and assistant nurses. BMC Health Serv Res.

[14] Stanley DJ, Meyer JP, Topolnytsky L (2005). Employee cynicism and resistance to organizational change. J Bus Psychol.

[15] Augustsson H, Morici BC, Hasson H, von Thiele Schwarz U, Schalling SK, Ingvarsson S, Wijk H, Roczniewska M, Nilsen P (2022). National governance of de-implementation of low-value care: a qualitative study in Sweden. Health Res Policy Syst.

[16] Kraus S, Schiavone F, Pluzhnikova A, Invernizzi AC (2021). Digital transformation in healthcare: analyzing the current state-of-research. J Bus Res.

[17] Van Velthoven MH, Cordon C (2019). Sustainable adoption of digital health innovations: perspectives from a stakeholder workshop. J Med Internet Res.

[18] Rogers E (2003). Diffusion of Innovations, 5th Edition.

[19] Eysenbach G (2004). Improving the quality of web surveys: the Checklist for Reporting Results of Internet E-Surveys (CHERRIES). J Med Internet Res.

[20] Bridge P D, Sawilowsky S S (1999). Increasing physicians' awareness of the impact of statistics on research outcomes: comparative power of the t-test and and Wilcoxon Rank-Sum test in small samples applied research. J Clin Epidemiol.

[21] Divine G, Norton H J, Hunt R, Dienemann J (2013). Statistical grand rounds: a review of analysis and sample size calculation considerations for Wilcoxon tests. Anesth Analg.

[22] Zumbo BD, Gadermann AM, Zeisser C (2007). Ordinal versions of coefficients alpha and theta for likert rating scales. J Mod Appl Stat Methods.

[23] Gadermann AM, Guhn M, Zumbo BD (2019). Estimating ordinal reliability for likert-type and ordinal item response data: a conceptual, empirical, and practical guide. Pract Assess Res Evaluation.

[24] Gagnon MP, Desmartis M, Labrecque M, Car J, Pagliari C, Pluye P, Frémont P, Gagnon J, Tremblay N, Légaré F (2012). Systematic review of factors influencing the adoption of information and communication technologies by healthcare professionals. J Med Syst.

[25] Almohtadi RM, Aldarabah IT (2021). University students' attitudes toward the formal integration of facebook in their education: investigation guided by rogers' attributes of innovation. World J Edu.

