# Supplementary material for: A Pragmatic Mapping of Perceptions and Use of Digital Information Systems in Primary Care in Sweden: Survey Study
Source: Interact J Med Res. 2023 Oct 25;12:e49973. doi: 10.2196/49973 (PMC10632913; doi:10.2196/49973)
Supplement: Multimedia Appendix 1 [file ijmr_v12i1e49973_app1.pdf]

Decision support in healthcare

Primary care data is reviewed regularly for quality assurance. This means that data from electronic health records and administrative data are processed to reveal key statistics and quality indicators. These statistics and indicators are often presented to the user as a digital dashboard, intended to inform and support quality work and administrative decisions. This survey aims to highlight how these decision support systems are used by primary care staff today.

This study stems from interdisciplinary research at Halmstad University in collaboration with business and the public sector. The work aims to increase understanding of innovative solutions that support information-driven care.

Research manager: Prof. Jens Nygren

Contact person: Anita Sant'Anna, PhD. (0735 09 30 33)

Privacy Policy: No personal information will be collected with this questionnaire. The link to this questionnaire may have been sent via email but your email address will not be tracked or stored. Participation is voluntary.

This questionnaire takes about 3 minutes. Do you want to continue?*

- ☐ Yes (goes on to next question)

What is your role in primary care? If more than one, state your main role.*

- ☐ Administrator, Operations Manager
- ☐ Physiotherapist, occupational therapist
- ☐ Registered nurse, district nurse
- ☐ Physician
- ☐ I do not work in primary care
- ☐ Other __________

*Obligatory question

Do you work at a private or public clinic?*

- ☐ Private
- ☐ Public

In which region do you work?*

[drop down list with all of Sweden's 21 Regions]

Primary Care Quality

Primary Care Quality [swe. *Primärvårdskvalitet*] is [Sweden's own] primary care quality system where you can easily follow your clinic's results in order to analyze and develop it. Care centers and rehab units that use Primary Care Quality can see data about their own operations and also follow up on the care of individual patients.

Do you have access to a digital tool where you can consult quality indicators and other statistics from health records and administrative data?*

- ☐ yes
- ☐ no
- ☐ I don't know

If yes, list which one(s).

(free text entry)

How often do you use this (these) tool(s)?*

- ☐ daily
- ☐ weekly
- ☐ monthly
- ☐ a few times a year
- ☐ very seldom or never
- ☐ we do not have access to a digital tool for quality assurance activities

*Obligatory question

How well do the statements below agree with your reason for using this(these) digital tool(s) today?*

	Completely agree	Partially agree	Neither or	Partly disagree	Completely disagree
To report to authorities.	☐	☐	☐	☐	☐
To plan budget and staffing.	☐	☐	☐	☐	☐
To design activities/processes to improve the quality of care.	☐	☐	☐	☐	☐
To identify individuals and groups at high risk or that incur high cost.	☐	☐	☐	☐	☐
To follow up specific patients' care journeys.	☐	☐	☐	☐	☐
Out of curiosity.	☐	☐	☐	☐	☐

Decision Support

Based on a large amount of data, decision support, unlike knowledge support, is expected to be able to provide patient-specific advice about risk factors, diagnosis, treatment, investigation and handling of individual patients. Within primary care, where many different conditions are managed, it is often related to screening and early diagnosis.

If a decision support system could be created in an instant, what decisions or processes should be targeted? How well do the statements below match your needs?*

	Completely agree	Partially agree	Neither or	Partly disagree	Completely disagree
To predict demand for care so we can better plan schedules and budgets.	☐	☐	☐	☐	☐
To obtain a list of likely diagnoses for a patient and suggestions for diagnostic tests.	☐	☐	☐	☐	☐
To receive suggestions for treatment options for a specific patient.	☐	☐	☐	☐	☐
To quickly identify high risk/cost patients as soon as they visit the clinic.	☐	☐	☐	☐	☐

*Obligatory question

	Completely agree	Partially agree	Neither or	Partly disagree	Completely disagree
To quickly identify high risk/cost patients before they visit the clinic.	☐	☐	☐	☐	☐
To remind patients to take their medication or follow their treatment.	☐	☐	☐	☐	☐
To help patients change their lifestyle (e.g. eating, exercising, smoking).	☐	☐	☐	☐	☐
To predict future disease development and care journey for a specific patient.	☐	☐	☐	☐	☐
To support taking joint decisions together with patients.	☐	☐	☐	☐	☐
To follow and interact with patients during their treatment.	☐	☐	☐	☐	☐

To what extent do you agree with the statements below?*

	Completely agree	Partially agree	Neither or	Partly disagree	Completely disagree
Digital decision support can improve the quality of my work.	☐	☐	☐	☐	☐
Digital decision support can improve the efficiency of my work.	☐	☐	☐	☐	☐
It is easy for me to learn to use digital decision support.	☐	☐	☐	☐	☐
It is easy for me to become skilled at using digital decision support.	☐	☐	☐	☐	☐
The use of digital decision support fits into our routines.	☐	☐	☐	☐	☐
The use of digital decision support is well suited to the way I like to work.	☐	☐	☐	☐	☐
I want to use digital decision support in the right way in my work.	☐	☐	☐	☐	☐

*Obligatory question

	Completely agree	Partially agree	Neither or	Partly disagree	Completely disagree
I want to use digital decision support on a trial basis long enough to see what they can do.	☐	☐	☐	☐	☐
I will use digital decision support when I see others using it in their work.	☐	☐	☐	☐	☐
I will use digital decision support in my work when I have knowledge of its clinical application.	☐	☐	☐	☐	☐
Using digital decision support in my work is a good idea.	☐	☐	☐	☐	☐
Using digital decision support in my work will make work more enjoyable.	☐	☐	☐	☐	☐

Thank you for your help!

You have now answered all the questions and can submit your answers. If you wish to go back and change your answer, you can click on "Back".

*Obligatory question
